# Co-designing policy, practice, and research directions for whole-school physical activity: towards sustainable culture change

**DOI:** 10.1186/s12966-026-01937-6

**Published:** 2026-06-17

**Authors:** Anna Chalkley, Zoe Helme, Ellen Silva, Shania Boom, Victoria Archbold, Daniel Bingham, Amika Singh, Antti Bloom, Caterina Pesce, Collin A. Webster, Edd Riley-Gibson, Esther van Sluijs, Gabriella McLoughlin, Geir Kåre Resaland, Jo Salmon, John Bartholomew, Juana Willumsen, Katrin Mägi, Lauren Clifford, Lawrence Foweather, Mathias Brekke Mandelid, Merike Kull, Natalie Lander, Nicole Nathan, Spyridoula Vazou, Stuart Fairclough, Tuija Tammelin, Andy Daly-Smith

**Affiliations:** 1 Institute for Health and Social Care, University of Bradford, Richmond Road, Bradford, BD7 1 DP UK; 2 Wolfson Centre for Applied Health Research, Bradford Royal Infirmary, Bradford, UK; 3 Bradford Institute for Health Research, Bradford Teaching Hospitals NHS Foundation Trust, Bradford, UK; 4 Mulier Instituut, Utrecht, The Netherlands; 5 School and Sport, Human Movement, Applied University of Windesheim, Zwolle, The Netherlands; 6 Finnish National Agency for Education, On the Move programme, Helsinki, Finland; 7 School of Health and Social Studies, Jamk University of Applied Sciences, Likes, Jyväskylä, Finland; 8 Department of Kinesiology, Michigan State University, East Lansing, Michigan, United States of America; 9 Department of Kinesiology, Texas A&M University-Corpus Christi, Corpus Christi, TX United States of America; 10 School of Psychological Sciences, College of Engineering, Science and Environment, The University of Newcastle, Callaghan, NSW Australia; 11 MRC Epidemiology Unit, University of Cambridge School of Clinical Medicine, Cambridge, UK; 12 Social and Behavioral Sciences, Barnett College of Public Health, Temple University, Philadelphia, United States of America; 13 Faculty of Education, Arts and Sports, Western Norway University of Applied Sciences, Røyrgata 6, Sogndal, 6856 Norway; 14 Institute for Physical Activity and Nutrition (IPAN), Deakin University, Geelong, VIC Australia; 15 Department of Kinesiology, Penn State University, Pennsylvania, United States of America; 16 World Health Organization, Geneva, Switzerland; 17 Institute of Sports Sciences and Physiotherapy, Department of Physical Education and Sports Didactics, University of Tartu, Tartu, Estonia; 18 Physical Activity Exchange, Research Institute for Sport and Exercise Sciences, Liverpool John Moores University, Liverpopol, UK; 19 Department of Pedagogy, Religion and Social Studies, Faculty of Education, Arts and Sports, Western Norway of Applied Sciences, Bergan, Norway; 20 National Centre of Implementation Science, College of Health, Medicine and Wellbeing, The University of Newcastle, Callaghan, NSW Australia; 21 Hunter Medical Research Institute (HMRI), New Lambton Heights, NSW Australia; 22 Department of Sport & Physical Activity, Edge Hill University, Ormskirk, UK

**Keywords:** Whole-school physical activity, Physical activity promotion, Children, Schools, Policy, Co-design, Context

## Abstract

**Background:**

Whole-school physical activity (WSPA) approaches are recognised globally as a key investment for increasing children’s activity levels. Their success depends on translating national policy into effective school practices through systems-level thinking and multi-stakeholder collaboration. This study aimed to use an experience-based co-design process to produce and prioritise future directions for WSPA policy, practice and research by engaging international stakeholders to identify key roles, needs, and actionable recommendations.

**Methods:**

The co-design process took place during a two-day international conference on Whole-School Physical Activity (#WSPA2024). Adopting a design thinking approach, 152 international stakeholders representing 16 countries engaged in two co-design workshops. Participants included professionals representing the policy (*n* = 64), practice (*n* = 26), or research (*n* = 62) sectors. Participants undertook tasks in either same stakeholder or mixed stakeholder groupings to explore and prioritise future needs for policy, practice, and research related to WSPA. All discussions were audio recorded, transcribed verbatim, and analysed using inductive thematic and content analysis. Prioritised actions were synthesised and recommendations to support the future of WSPA were drafted. Stakeholders reviewed the drafted recommendations and proposed modifications using an online questionnaire.

**Results:**

Twenty-four recommendations (10 for policymakers, eight for practitioners, and six for researchers) to prioritise and plan for WSPA were developed. These are represented by six interlinked themes reflecting conditions for change: systems thinking, partner engagement and collaboration, equity and inclusion, evidence-based and action orientated, knowledge mobilisation, support and capacity building.

**Conclusions:**

WSPA approaches should be viewed as a long-term systems change agenda requiring aligned policy, adaptable practice, and innovative research. The WSPA recommendations provide a platform for multi-sector collaboration, co-production, and investment in scalable, context-sensitive solutions.

**Supplementary Information:**

The online version contains supplementary material available at https://doi.org/10.1186/s12966-026-01937-6.

## Background

Children’s physical inactivity poses a ‘wicked problem’ [1]. Despite widespread recognition of its importance, population level physical inactivity is increasing [2] and has been described as a key public health priority [3]. Globally, over 80% of school-going adolescents (aged 11–17) worldwide do not meet recommended physical activity guidelines [4] of accumulating an average of one hour of moderate to vigorous physical activity (MVPA) each day across the week [5]. Addressing this challenge is particularly critical for children who stand to receive the triple benefits (today, into adulthood, and for the next generation) that positive lifestyle behaviours can yield [6].

Physical activity is shaped and sustained by the complex system in which it takes place [7]. Large scale solutions, including a range of systems-based actions, are needed to support population wide shifts in physical activity across multiple settings, including schools and education-based settings [8]. Whole-school physical activity (WSPA) approaches are recognised internationally as one of the eight best investments, with the potential-when implemented at scale-to significantly increase activity levels among children [9]. These approaches require alignment across school-level policies, environments, stakeholders, and physical activity opportunities [10]. However, their success depends on the effective translation of national policy actions into daily school practices that can meaningfully increase activity [11, 12].

It is widely recognised that schools are a valuable and pragmatic setting with the potential to share positive health behaviours among children and young people [13]. The importance of schools as key focal points for policy action is demonstrated in several high-level documents relating to sport and physical activity, for example the Global Action Plan on Physical Activity 2018–2030 [8] and the International Society for Physical Activity and Health’s Eight Investments that work for physical activity [9] However, in practice, data shows there is large variability in pupil’s physical activity between schools [14] and identifying whether children have compatible opportunities within school is important in mitigating health disparities in underserved populations [15].

A persistent challenge lies in the disconnect between the national development of policies and their subnational implementation to support physical activity [16]. This is because schools, as complex adaptive organisations embedded within wider social systems [17], often face constraints that limit individual teacher agency and shape how policies or practices are enacted [18–20]. As a result, although single- or multi-component school-based interventions are well evidenced, review-level research suggests they are largely ineffective when replicated or scaled in real-world contexts due to limited stakeholder engagement [21].

Addressing these challenges requires systems-level thinking and the need for engagement of a wide range of stakeholders working with(in) education settings to ensure that the perspectives of those affected by policies and interventions are valued and prioritised [22]. WSPA initiatives, such as the Comprehensive School Physical Activity Program in the United States of America [23], Estonia’s Schools in Motion [24] and Creating Active Schools (CAS) in the UK [10], illustrate the need to align education and health policy, practice, and research, requiring a transdisciplinary approach that integrates health and educational disciplines [25]. Such strategies represent a shift towards bridging the health and education nexus, though their success ultimately relies on political will for implementation and support [26].

Both bottom-up and top-down approaches must be galvanised to drive change in public health [27]. Crucially, these need to be supported by vertical and horizontal communication channels to strengthen collaboration and participation-factors that policymakers frequently overlook [28]. For national policies relating to school-based physical activity to succeed, political support, cross-sectoral collaboration, and engagement of stakeholders at multiple levels are essential [29]. Together, these factors can improve understanding, enhance policy delivery, and provide the sustained support needed for effective implementation [30]. Therefore, the aim of this study was to co-design and prioritise future directions for WSPA policy, practice and research through international multi-stakeholder engagement. Specifically, we sought to: examine stakeholders’ perspectives on their roles in improving WSPA; explore and prioritise future needs for policy, practice and research in this area, and develop actionable recommendations through participatory co-design methods.

## Methods

The co-design process took place during a two-day international conference on Whole-School Physical Activity (#WSPA2024), held from 17 to 19 June 2024 (www.wspa2024.co.uk). The University of Bradford, the Wolfson Centre for Applied Health Research, and the Yorkshire Sport Foundation collaborated to host the conference which aimed to share learning, influence leaders, and shape future policy. It showcased eight leading WSPA approaches from around the world, advancing understanding of what works and drawing insights from those delivering and supporting such programmes. Uniquely, #WSPA2024 brought together a diverse mix of multisector representatives including policymakers, national governing organisations, school-based practitioners, teacher educators, researchers and evaluators from 24 countries.

### Study design

This study adopted a qualitative co-production process which sought to capitalise on the breadth and depth of #WSPA2024 delegates’ expertise through the integration of two 90-minute co-design workshops. The purpose of the workshops was to facilitate a shared understanding of the role that conference delegates, and their contemporaries can play in improving WSPA and identify what the future priorities are for policy, practice and research related to this area. The study design and protocol was based on previous studies conducted by the authorship team using a similar approach, see [10, 25].

Ethical approval was granted by the Chair of Humanities, Social, and Health Sciences Research Ethics Panel at the University of Bradford (protocol code E1029, date of approval: May 20, 2024). To enhance transparency, this study was reported in accordance with the Standards for Reporting Qualitative Research (SRQR) [31].

### Sampling and participants

All registered delegates (*n* = 261) were notified of the intention of the workshops and their purpose prior to the conference and invited to participate in the study by email. Delegates were allocated a stakeholder group based on information regarding their job role, provided at the time of registering, and the expectation of them being able to provide practical, operational and conceptual knowledge of WSPA [32]. Three stakeholder groupings were formed:

1. Policymakers – included civil servants affiliated within governmental departments and employees of state agencies and public bodies with responsibility for developing/advising on local, regional, national government policy and decision making and are potential users of research to improve practice (e.g. Strategic lead, Education Manager, Active Travel Officer).
2. Practitioners – included anyone working with/in the school setting specifically in relation to the strategic development and/or delivery of physical activity opportunities across the school day and beyond (e.g. Headteacher, Teacher, PE lead).
3. Researchers - included those who systematically collect, organize and synthesise research and evaluation data (e.g. Research Fellow, Lecturer, PhD student).

During the conference registration, all delegates received an information sheet and were asked to complete and return a signed consent form. At the start of each workshop, participants were advised that discussions would be recorded and used for data collection and that they were free to withdraw at any time without it affecting their conference participation.

### Data collection

The co-design workshops adopted an evidence-based co-design approach informed by design thinking philosophy [33] Co-design involves active collaboration between researchers, designers, developers and end users, who are recognised as “experts in their own experiences” [34]. Drawing on existing knowledge and newly generated insights, stakeholders are supported to develop intervention principles, with the process designed to encourage creative thinking. The workshops were held on the second day of the conference to ensure participants had a strong understanding of WSPA and that networking, learning, and differing perspectives could feed into discussions. The co-design workshops comprised two 90-minute workshops which included a series of interactive tasks to answer key questions intended to prompt discussion, critically reflect, and generate ideas. The workshops were conducted in English facilitated by University of Bradford staff trained in experience-based co-design and participatory methods. The facilitators assisted by conference volunteers, encouraged participants to articulate, and elaborate on, their thoughts by using inclusive communication methods, such as requesting that native speakers reduce speaking speed, avoid idioms, and encouraging participants to indicate when they did not understand.

#### Workshop 1: Same stakeholder groups

Participants were first grouped by disciplinary background: policymakers, practitioners and researchers. To ensure a breadth of perspectives, attention was given to ensure each group included individuals with varied roles and from multiple countries. Owing to the size of the policymaker cohort, this group was divided into two subgroups: (1) local and regional policymakers and (2) national and international policymakers. Once in the designated room, participants were randomly divided into smaller groups of six-to-eight per table. Each room was led by a senior facilitator with support from a minimum of three research assistants. Concurrent discussions during the workshops were audio recorded using dictaphones placed on each table.

Participants were asked to discuss the first question “*What do you think are the key ingredients for an optimal whole school physical activity approach*?” before discussing and capturing their tacit knowledge on post-it notes. These were subsequently reviewed by the other groups during a period of sharing and reflection, and delegates were given the opportunity to evolve their responses before being asked to address the second question “*What are the priorities for your stakeholder group in helping schools to achieve a whole-school physical activity approach*?”.

At the end of the workshop, participants were asked to write a synopsis of their discussions and take a photo of their post its to refer to for the second session. Each participant was then allocated a location for the second (mixed stakeholder) workshop by the lead facilitator of the room. This was based on their perceived level of engagement and the quality of discussions held in workshop 1. For example, this included monitoring both verbal and non-verbal cues, assessing interactional dynamics, and observing how participants responded to the subject matter.

#### Workshop 2: Mixed stakeholders

Participants were asked to discuss a third question “*If you were writing recommendations for future research*,* policy and practice*,* what would be your main asks*?”. Participants were encouraged to share, discuss and capture their tacit knowledge on post-it notes. These were subsequently reviewed by the other groups during a period of sharing and reflection before being asked to evolve and rank their ideas from most important to least important, creating a top six for each discipline (research, policy and practice).

### Data analysis

Data were analysed using inductive thematic and content analysis to gain a more comprehensive understanding of data by integrating qualitative insights with quantitative trends [35]. This combined approach allowed for the identification of in-depth themes through thematic analysis while simultaneously quantifying their frequency, relevance, or other patterns using content analysis, creating a richer, more complete picture from the data.

Group discussions, post-it notes and ranked priorities were transcribed verbatim into Microsoft Word. Data were analysed by authors ZH and AC, with the aid of Miro to synthesise data into broad concepts and patterns using an open coding frame based on inference and interpretation [36]. Both, ZH and AC have undergone training in qualitative methods, including data analysis, and have significant experience of conducting qualitative research and/or working in the physical activity and public health sector.

Using an inductive approach and an open coding frame to allow broad concepts and patterns to be identified, data were coded into themes based on meanings and impressions at both the latent and semantic level [37]. Interpretations of the post-it notes and priorities for action were supported by participants’ quotes from the transcripts. Thematic codes were grouped into larger categories or subthemes and subsequently main themes. Candidate themes were presented to author ADS and interpretations were openly discussed and challenged by critically probing for explanations to achieve a final consensus.

Once agreed, recommendations were then scored on a sliding scale according to their participant ranking. For example, if they had been ranked 1st, the recommendation was given six points, 2nd, five points, 3rd, four points, and so on. If recommendations were not ranked (e.g., a recommendation was not placed 1–6, or a group had written more than six and it was not ranked within their top six), they were still aligned to a theme (i.e. through the wording or terminology used) but were given a score of zero. The total scores for each theme and sub-theme were calculated using the total rank scores of each recommendation.

Commonality was assessed and similar recommendations were grouped to avoid duplication and increase coherence. Final recommendations included themes which were not only ranked as having the highest priority but were also mentioned by more than one group. These were then synthesized and translated into draft recommendations whilst retaining the original wording used by participants.

**Fig. 1:**
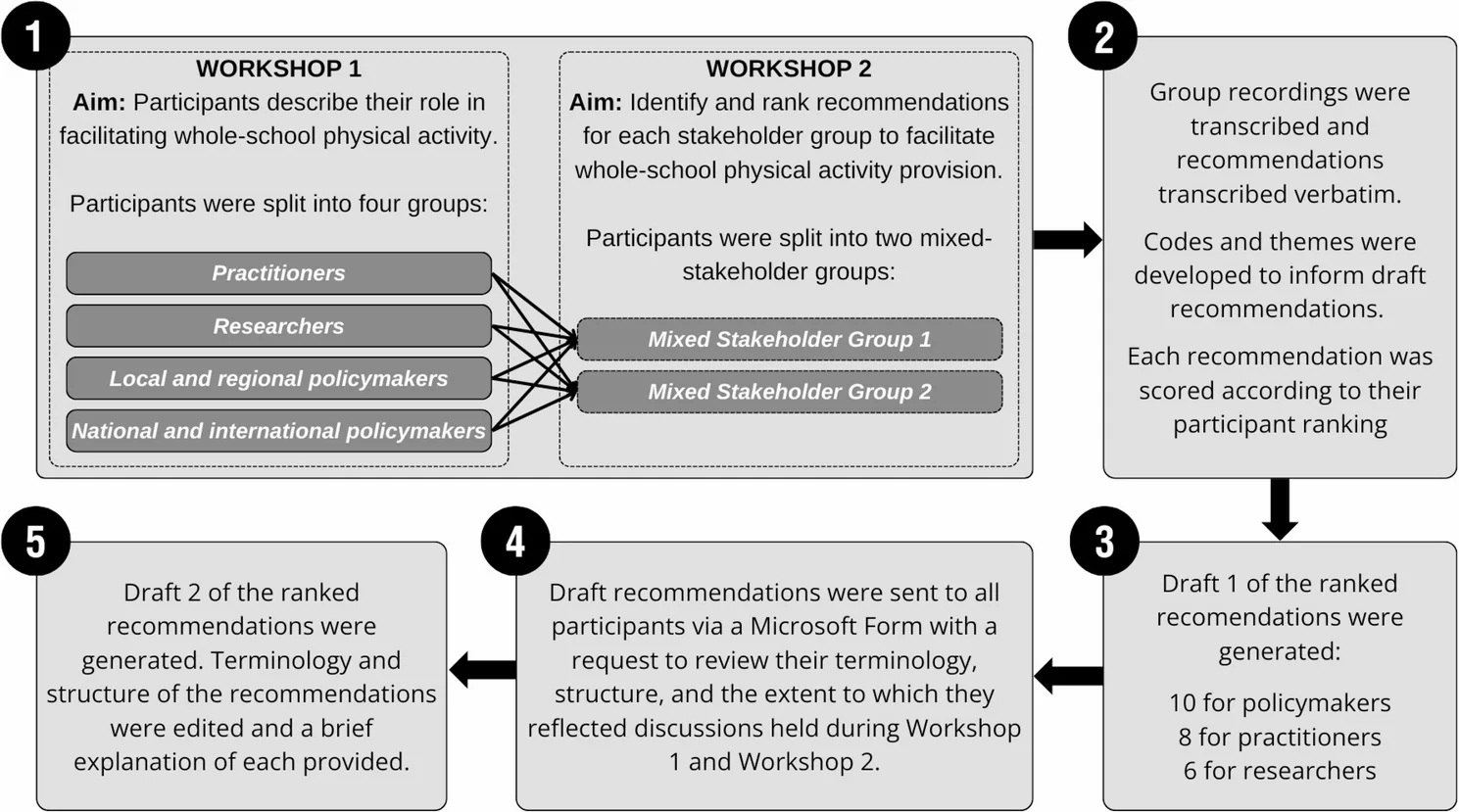
A summary of the co-design process used for the creation of the WSPA recommendations

A refinement process was subsequently undertaken remotely, six months after the event. The draft recommendations were shared with all of the participants, with each recommendation presented twice; the first for participants to reflect on the extent to which they aligned with their conversations during the workshop(s), and the second to allow participants to give feedback on the recommendation’s wording, terminology, and structure. A link to a Microsoft Form was emailed to all participants and remained open for three weeks, with a reminder email sent after two weeks. Thirty responses were received, representing a response rate of ~ 26%. A summary of the data collection process is provided in Fig. 1 below.

## Results

Of the 261 conference delegates, 152 (58.24%) agreed to participate in the study and engaged in one or both of the co-design workshops. Participant dropout occurred between the workshops due to participants leaving the conference due to travel or work commitments. Therefore, findings are based on the 127 participants who took part in the first workshop and the 113 participants who participated in the second.

Whilst there was parity in terms of participant numbers across the policymakers and researchers’ groupings, there were fewer practice-based participants (64, 62, and 26 respectively), however this was proportionate to the conference delegation as a whole. In addition, the practitioner group almost entirely consisted of practitioners from the UK, whereas the other stakeholder groups were represented by multiple countries. A summary of participants by participant group is provided below in Table 1.

**Table 1:** Summary of participant characteristics

Category	Total Participants				Workshop 1				Workshop 2			
	Po (*n*, %)	*R* (*n*, %)	Pr (*n*, %)	Total (*n*, %)	Po (*n*, %)	*R* (*n*, %)	Pr (*n*, %)	Total (*n*, %)	Po (*n*, %)	*R* (*n*, %)	Pr (*n*, %)	Total (*n*, %)
Total sample	64 (42%)	62 (41%)	26 (17%)	152 (100%)	49 (39%)	53 (42%)	26 (20%)	127 (100%)	50 (44%)	42 (37%)	21 (19%)	113 (100%)
Gender												
Male	26 (41%)	24 (39%)	15 (58%)	65 (43%)	23 (47%)	21 (40%)	15 (58%)	58 (46%)	24 (48%)	17 (41%)	10 (48%)	51 (45%)
Female	37 (58%)	38 (61%)	11 (42%)	87 (57%)	26 (53%)	32 (60%)	11 (42%)	70 (55%)	26 (52%)	25 (60%)	11 (52%)	62 (55%)
Country												
Australia	—	5 (8%)	—	5 (3%)	—	5 (9%)	—	5 (4%)	—	5 (12%)	—	5 (4%)
Belgium	—	1 (2%)	—	1 (1%)	—	—	—	—	—	1 (2%)	—	1 (1%)
Canada	2 (3%)	—	—	2 (1%)	1 (2%)	—	—	1 (1%)	2 (4%)	—	—	2 (2%)
Chile	—	1 (2%)	—	1 (1%)	—	1 (2%)	—	1 (1%)	10 (20%)	—	—	—
Czech Republic	—	1 (2%)	—	1 (1%)	—	1 (2%)	—	1 (1%)	—	1 (2%)	—	1 (1%)
Denmark	10 (16%)	2 (3%)	—	12 (8%)	6 (12%)	2 (4%)	—	8 (6%)	—	—	—	10 (9%)
Estonia	—	4 (6%)	1 (4%)	5 (3%)	—	4 (7%)	1 (4%)	5 (4%)	—	3 (7%)	1 (5%)	4 (4%)
Finland	—	1 (2%)	—	1 (1%)	—	1 (2%)	—	1 (1%)	—	—	—	—
Italy	—	1 (2%)	—	1 (1%)	—	1 (2%)	—	1 (1%)	—	1 (2%)	—	1 (1%)
Malta	—	1 (2%)	—	1 (1%)	—	1 (2%)	—	1 (1%)	—	1 (2%)	—	1 (1%)
Netherlands	2 (3%)	2 (3%)	—	4 (3%)	2 (4%)	1 (2%)	—	3 (2%)	2 (4%)	2 (5%)	—	4 (4%)
Norway	—	3 (5%)	—	3 (2%)	—	—	—	—	—	3 (7%)	—	3 (3%)
Portugal	—	1 (2%)	—	1 (1%)	—	1 (2%)	—	1 (1%)	—	1 (2%)	—	1 (1%)
Switzerland	2 (3%)	—	—	2 (1%)	1 (2%)	—	—	1 (1%)	1 (2%)	—	—	1 (1%)
UK	47 (73%)	35 (57%)	25 (96%)	107 (70%)	38 (78%)	32 (60%)	25 (96%)	95 (75%)	34 (68%)	20 (48%)	20 (95%)	74 (66%)
USA	1 (2%)	4 (6%)	—	5 (3%)	1 (2%)	3 (6%)	—	4 (3%)	1 (2%)	4 (10%)	—	5 (4%)

*Po* Policy, *R* Research, *Pr* Practitioners

Numbers are counts (n) with percentages (%) of total in each group. Zeros shown as dashes (—)

Following the refinement process, 24 unique recommendations were developed (10 for policymakers, eight for practitioners, and six for researchers). Together, these recommendations call for a long-term, system-wide approach that integrates policy, practice and research to create healthy school environments.

The policy recommendations call for clear national standards, strong accountability mechanisms, and long-term investment to embed physical activity within education systems. They emphasise understanding local school contexts, fostering cross-sector collaboration, and involving all stakeholders in policy design and review. Key asks include updating curricula, assessments, and teacher training to integrate physical activity and physical literacy across the school day; establishing sustainable funding frameworks; and creating monitoring systems that capture meaningful indicators of physical activity, health, engagement and well-being. Overall, the policies advocate a whole-system, evidence-informed approach that supports relevance, equity, and long-term cultural change.

The practice recommendations centre on building school-wide capacity, culture, and ownership for physical activity. They encourage regular context assessment, stakeholder engagement, and shared responsibility through supportive leadership and collaborative planning. Key priorities include empowering pupils’ voices, embedding physical activity into school policies and routines, and supporting teachers with ongoing professional development. Establishing communities of practice and recognising that sustainable change occurs gradually through small steps are also essential. Overall, these recommendations focus on equipping schools with the skills, structures, and relationships needed to make physical activity a valued and integrated part of daily school life.

**Fig. 2:**
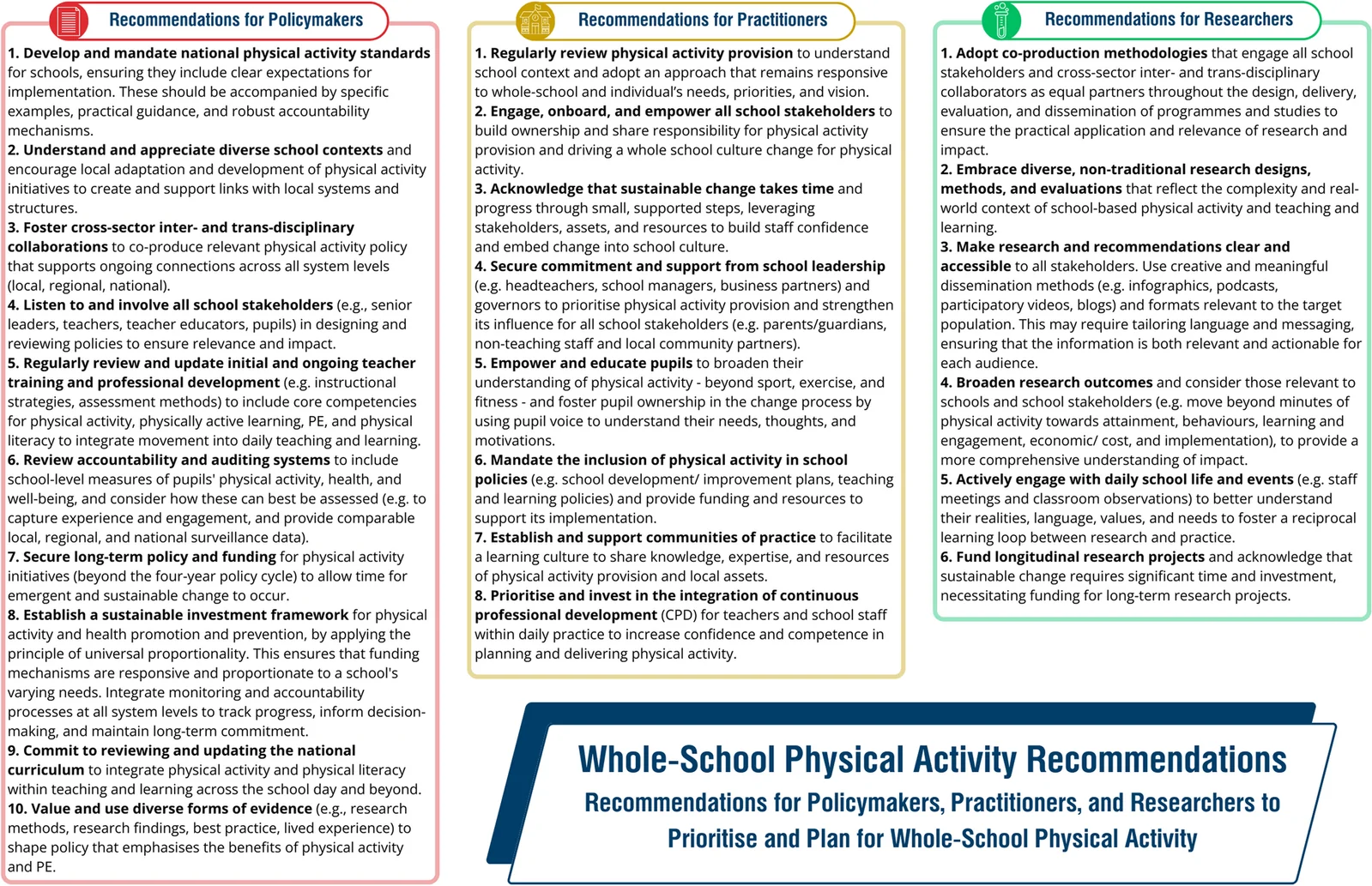
A summary of the Whole-School Physical Activity Recomendations

The research recommendations advocate for co-produced, practice-relevant studies using diverse, real-world methodologies that reflect the complexity of school environments. They call for accessible, meaningful dissemination tailored to different audiences, and for expanding research outcomes beyond traditional metrics to include learning, engagement, economic, and implementation factors. Researchers are encouraged to immerse themselves in school settings to better understand context and strengthen the research–practice feedback loop. Finally, long-term funding for longitudinal studies is essential to capture the sustained, systemic nature of whole-school physical activity change. See Fig. 2 for a copy of the recommendations, presented in order of priority.

The results presented here are based on the thematic analysis of participant discussions, used to develop the recommendations relating to the development of sustainable WSPA approaches. Six interrelated themes, representing conditions for change relating to WSPA, were identified and are presented below supported by participant quotations.

### Systems thinking

Participants consistently emphasised the need to position a WSPA approach as a systems-level intervention that recognises the complex and multilayered nature of school-based physical activity. WSPA was understood as both shaping and being shaped by the wider system in which it operates. This includes factors such as senior leadership, national curriculum requirements, school culture, physical and social environments and partnerships. A systems framing was therefore seen as essential for moving beyond fragmented and initiative-based delivery towards a more holistic and place-sensitive approaches, something participants felt had often been lacking:

*“…consider the school as part of the system*,* which are the components. And then consider how schools fit into the wider system. Yes*,* that’s interesting.”* (Table 6, Researcher, England).

From this perspective, sustainable change was thought to be more likely when attention is paid to the interactions between policies, practices and relationships across multiple levels of influence. Participants reflected that adopting a systems approach would also require a shift in how WSPA research is conducted. They called for methods and frameworks capable of ensuring complexity, including participatory, interdisciplinary and longitudinal approaches that move beyond linear cause and effect models:

*“The approach is inherently complex and messy*,* right? So I don’t think we’ll ever get anywhere until we can zoom out and use methods and support methods that can capture it*,* but that requires*,* like*,* a high level of discomfort*,* I think*,* on the part of researchers and on the part of reacting bodies of those who are on the receiving end of research*,* I think it’s harder to do this and might be seen as nuts.”* (Table 3, Policymaker, England).

Such approaches were seen as better suited to capturing the realities of how change occurs in schools and to designing interventions that are both effective and feasible in real-world contexts. Participants also highlighted the need for research agendas to prioritise outcomes that matter to children, teachers and policymakers alike, including broader and more holistic indicators such as belonging, relatedness and mental health and wellbeing, rather than focussing on minutes of MVPA.

At the policy level, systems thinking was most often discussed in relation to the need to bridge siloed ways of working across education, health and community sectors. For example, one participant remarked:

“*We’re never going to get the system back*,* the whole system approach*,* are we*,* without reviewing what’s going on first and collaborating across sectors”* (Table 7, Policymaker, England).

Participants noted tensions between existing standards accountability systems and funding streams for physical activity, PE and school sport, which often create confusion and inconsistency, as one participant reflected:

*“the school system is so complex*,* with so many different levels*,* and with different responsibilities. And that’s*,* I think*,* a major challenge on policy level*,* to create a common understanding of what is this [WSPA] and why we do it” (Table 4*,* Policymaker*,* Denmark).*

A systems approach was therefore viewed as a way to better align strategies across sectors, promote clearer guidance, and support longer- term investment. This included moving away from short term policy cycles towards sustained funding frameworks that recognise physical activity as a public health prevention strategy rather than an optional enrichment activity where physical activity is treated as an “add on”. Consequently, participants argued strongly for funding models grounded in proportionate universalism, whereby resources are targeted according to need.

At the school level, systems thinking was seen as providing the stability and structure needed to embed physical activity into everyday practice. By aligning policies, environments, stakeholders and opportunities, schools could integrate physical activity into the fabric of school life, supporting not only health outcomes but also educational priorities such as attainment, engagement and wellbeing.

### Partner engagement and collaboration

Participants widely agreed that systems change cannot be achieved by any single organisation or sector acting alone. Meaningful engagement and collaboration across stakeholders was therefore seen as essential conditions for sustainable WSPA approaches, as one participant shared:

“*Look at the wider picture*,* speaking as a local authority*,* that what we do is we don’t just work with the active bit. So we link*,* we link in with public health*,* with leisure*,* with primary care network*,* with the NHS*,* so that it’s a really wide scope*” (Table 9, Policymaker, England).

Collaboration was understood as a way of bringing together diverse expertise, resources and perspectives to address the multiple factors shaping physical activity behaviours. Importantly, participants emphasised that engagement should be embedded across all stages of the research process, from design through to delivery and evaluation, to ensure relevance, feasibility, and real-world impact.

*“There’s something about involving schools or organizations in designing the research in the first place and supporting the grant application process. So more involved*,* more stakeholder involvement at that early stage. It has to be meaningful stakeholder involvement*,* so not tokenistic.” (Table 15*,* Policymaker*,* Scotland).*

This approach was believed to support real-time learning, improve translation of evidence into practice and strengthen the connection between research, policy and implementation. Policymakers were viewed as particularly important partners when engaged as collaborators rather than solely as funders or regulators, enabling better policy alignment and more informed decision making.

For practitioners, collaboration was seen as central to building confidence, capacity and sustainability. Working with partners enabled access to specialist expertise, professional learning opportunities and peer support networks. Participants highlighted the value of school- to school learning and professional networks in reducing isolation and enabling adaptation of successful approaches across contexts as one participant shared:

“*Because you’re always going to have in primary school teachers that aren’t confident doing it*,* yeah. You’ll always have someone who goes to their support network to look for help and make connections and relationships with them. And that might be another school*,* yeah*,* because*,* rather than them working in silo and trying to do everything by themselves and make mistakes*,* go look at other schools. So be part of a network*,* I guess*,* of some sorts.*” (Table 7, Practitioner, England).

Co-production was frequently highlighted, particularly by research-based stakeholders, as a way of avoiding top-down mechanistic interventions that fail to fit school realities, as one participant described:

“*I think a lot of us are guilty of doing that*,* dreaming up something that we think will work*,* making a case for it*,* and off we pop*”. (Table 10, Policymaker, England).

Across all groups, participants underscored the importance of engaging a full spectrum of stakeholders, including pupils, teachers, senior leaders, parents and partners from the voluntary, private and charitable sectors across all levels of the system.

### Equity and inclusion

Equity and inclusion were viewed as fundamental principles that should guide decision making, implementation and evaluation of WSPA. Participants shared a strong belief that schools have a critical role in promoting fairness in health and wellbeing outcomes but that this requires an explicit focus on addressing inequalities.

For WSPA programmes to effectively address disparities, participants shared that they must be designed and implemented to remove the specific barriers that prevent some children from participating fully. At a school level this included embedding physical activity within school improvement plans and policies, integrating movement across the curriculum through physically active learning, and ensuring that opportunities are culturally and contextually relevant.

At the national level, participants emphasised the importance of cross-sector collaboration to avoid duplication, align messaging and ensure a more equitable distribution of resources, particularly prioritising schools and communities facing the greatest disadvantage.

“*The question is whether*,* whether this needs to be more laser focused on tackling inequalities*,* because there’s never enough like the what we always seem to try and do at the policy level is give everybody the same amount of money. And it shouldn’t be like that.*” (Table 3, Policymaker, England).

Participants also highlighted the need to include underrepresented voices in policymaking and research, including practitioners and pupils. This extended to using flexible and inclusive data collection methods and being attentive to language used when communicating with stakeholders, to avoid creating additional barriers.

“*They [Education Endowment Foundation] do research pieces*,* but then they put them into*,* like*,* toolkits and kind of*,* like*,* easily digestible reports. So kind of our*,* one of our first places teachers to go to is that is there. And I think it comes back to time as well*,* like*,* as a class teacher*,* we don’t*,* I don’t have time to go and read research.*” (Table 3, Practitioner, England).

Pupil voice was seen as especially important in shifting perceptions of physical activity beyond sport or fitness and fostering a sense of ownership. Involving pupils in decision making around the design and delivery of provision was viewed as central to developing inclusive approaches that reflect the diversity of school communities, this was reflected by one participant who said:

*“It kind of starts to talk about like inequality and how do schools overcome inequality? By the way*,* that like the conversation that they’re having with children*,* giving children their own voice*,* that all kind of comes together in one area*,* almost*.” (Table 3, Policymaker, England).

### Evidence-based and action orientated

Participants reflected the importance of grounding WSPA strategies in evidence, while recognising that evidence should be broadly defined to include practitioner expertise and the needs and preferences of children and schools. This required valuing multiple forms of evidence and diversifying research methods to capture complexity and context.

“*What works well to me as an academic*,* might be different from people who are practitioners*,* and I think they’re sometimes there’s also about that shared language and commonality and thinking about actually getting lots of different perspectives on the same thing*,* and them all being equally valuable*,* you know?*” (Table 20, Researcher, Scotland).

There was a clear call to move beyond reliance on traditional approaches such as randomised controlled trials towards approaches such as realist evaluation, systems science and participatory methods that better reflect real world settings. Pragmatic evaluation approaches were also seen as important in supporting schools to monitor implementation and inform decision making.

“*I think that teachers are using (lived experience) to gather their own evidence*,* and it’s not the same way that we would think of something being evidence based*,* but it’s evidence based actually on the ground. It’s context specific. So it’s just as valuable to schools*.” (Table 5, Practitioner, England).

Effective communication and dissemination were viewed as critical for translating evidence into action. Participants highlighted the need for transparent reporting and the use of accessible formats such as infographics, videos and digital platforms like social media, blogs and podcasts to reach non-academic audiences.

“*If I could watch a two-minute video and get the summary points that I get to understand*,* that’s much more user friendly*.” (Table 3, Policymaker, Canada).

Finally, participants argued that policy must be evidence led to build credibility and secure buy-in. Longitudinal research demonstrating cultural, behavioural and organisational change as well as broader child and school level outcomes was seen as essential for shifting the status quo.

### Knowledge mobilisation

Knowledge mobilization emerged as a key mechanism for bridging the gap between research, policy and practice. Participants emphasised the importance of strong partnerships to collaboratively translate evidence into action and ensure that WSPA is supported through coherent national and local policy.

There was widespread agreement that research evidence, professional expertise and lived experience should be valued equally and shared across the system. Mechanisms such as collaboration and co-design were seen as central to enabling two-way knowledge exchange and developing locally relevant solutions.

“*We need to go up to schools*,* see what happens in schools*,* bring that with us back and kind of co-create opportunities for being more physically active*,* I think. And that’s why I’m saying you can’t just rely on evidence. We need to rely on research. We need to rely on experimental knowledge and practice*.” (Table 10, Researcher, Norway).

Embedding learning into everyday practice was frequently discussed, particularly through professional learning communities and peer-to-peer exchange. These structures were viewed as ways to accelerate knowledge mobilisation and enable evidence and practice to inform each other in real time. The use of embedded researchers was highlighted as one approach to fostering a culture of enquiry within schools.

### Support and capacity building

This theme highlights the need for sustained investment in people, systems, and structures to support the implementation and long-term sustainability of WSPA. Participants consistently pointed to the limitations of short-term funding and policy cycles, calling instead for long-term commitments that provide clear, practical guidance and direct resources to where they are most needed.

“*You’d like to think that policy might direct attention to the most important things to implement*,* what those high leverage strategies are based on evidence? Yeah*,* but I suppose then that leaves a bit of a vacuum as to what does that look like across different contexts. And obviously policy has to stay vague and ambiguous because it has to be so broad*,* but I think it would be useful if there was like a range of more tangible or concrete examples to build a picture. What does this look like in reality? So if you’re a practitioner who might be struggling to grasp something that’s quite abstract*,* it can help you too.*” (Table 16, Researcher, England).

Without adequate support, school staff were seen as lacking the confidence, competence, and capacity to embed physical activity amid competing pressures.

“*And actually*,* if you are busy and you’re stressed and you’ve got other concerns about attainment and Ofsted and all that stuff that’s tricky to do*,* and you need permission and time and space to do that and support.*” (Table 11, Researcher, England).

Participants therefore emphasised the importance of strengthening initial teacher education and providing ongoing professional development to support reflective practice and communities of practice. Distributed leadership and shared responsibility across stakeholders were viewed as essential for culture change, requiring time, small-step approaches, and sustained buy-in.

Reciprocal learning was also identified as a priority, with participants arguing that schools and policymakers should help shape research agendas in the same way that research informs practice. Building research capacity through participatory, co-produced approaches was seen as central to achieving this vision and ensuring that evidence remains timely, relevant, and responsive to the evolving realities of school systems.

## Discussion

**Fig. 3:**
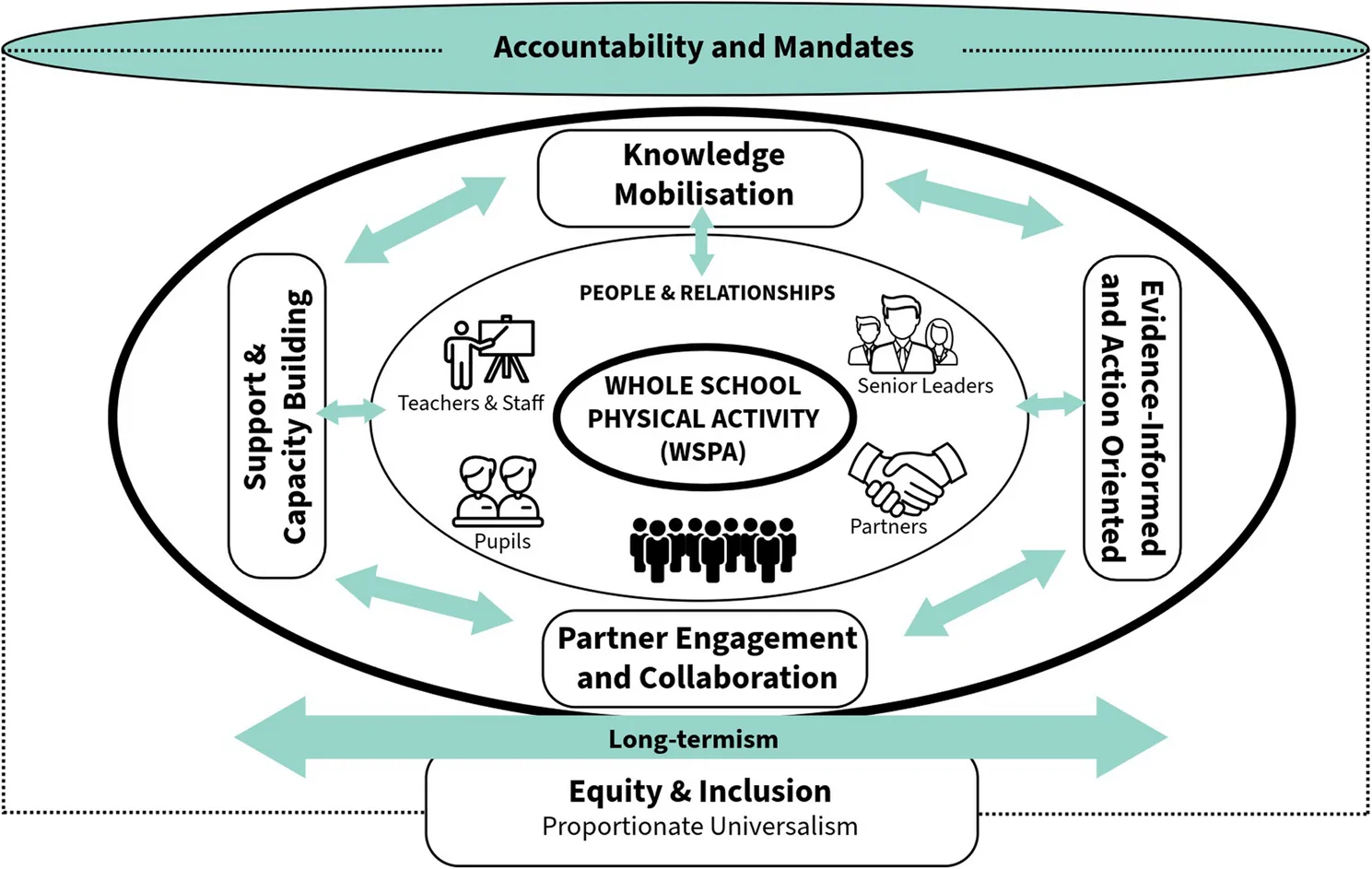
An empirically-informed systems model of whole-school physical activity (WSPA)

This study advances not only the WSPA field but also contributes to the wider literature on school-based and setting-based health interventions by reframing implementation as a question of system conditions rather than programme components. While much of the existing evidence base across school health, public health, and prevention science continues to focus on intervention effectiveness, fidelity, and scale-up, our findings align with a growing systems movement that recognises schools as complex adaptive organisations embedded within wider policy, accountability, and community structures. By foregrounding relational infrastructure, equity, knowledge mobilisation, and long-term capacity building as core mechanisms of change, this work extends beyond WSPA to inform how school-based interventions more broadly can move from short-term projects to sustained cultural transformation within education systems. In doing so, this work calls for those within public health and education to address children’s physical activity using the 24 recommendations for systems-oriented, equity-driven, and implementation-focused approaches that better reflect real-world complexity. The themes are presented as an empirically informed systems model of WSPA (see Fig. 3).

The unique contribution of the recommendations lies in their systems thinking orientation and the conceptualisation of WSPA as an interconnected system where actions should be integrated across multiple domains (i.e. policy, practice and research [1]). Consequently, the model is presented as a nested systems model with WSPA at the core, representing both an outcome of the system and an active layer that reshapes it. The dynamic interactions are represented by the bi-directional arrows between all layers of the system and the feedback loops. These help to reinforce that outcomes, and change, emerge over time.

Equity, inclusion and long-termism sit beneath and across the entirety of the model as foundational principles shaping how physical activity is planned, implemented and sustained (Policy recommendations 7 & 8, Research recommendation 6). Their position reinforces the argument that WSPA requires long-term investment and commitment to further our understanding of schools as embedded within complex adaptive systems, and the need to continually evolve practice (Practice recommendation 3). Our findings resonate with critiques of ‘projectified’ public health approaches that prioritise outcomes over durable system change [27]. Without this, system change would remain fragile and uneven.

From a systems perspective, inequalities in physical activity arise through the interaction of policies, environments, resources, social norms, and power relations. Without an explicit equity lens, WSPA approaches risk reinforcing existing disparities in access, participation, and benefit [38]. Consequently, the recommendations call for equity and inclusion to guide priorities, partnerships, and evidence use, aligning with principles of proportionate universalism, whereby universal provision is combined with targeted support according to need [39] (Policy Recommendation 8). For WSPA this includes centring pupil and practitioner voices, valuing lived experience alongside research evidence, and designing flexible frameworks that can be adapted across diverse school contexts (Policy recommendation 10, Practice recommendation 5, Research recommendation 2).

Similarly, an equity-informed approach to provision would help broaden stakeholders’ understanding of physical activity beyond sport to include movement, play, and wellbeing, integration of activity across the curriculum, and supports inclusive pedagogies and environments [40] (Practice recommendation 5). Finally, embedding equity within accountability and learning processes further ensures that WSPA delivers fair and sustainable benefits for all children and young people (Policy recommendation 6, Practice recommendation 1).

Immediately surrounding the WSPA layer is a relational layer representing how sustainable WSPA is socially produced by the different agents and school stakeholders which operate within it (i.e. school leadership, collaborators and system partners). The centrality of relationships, leadership and capacity building within the findings aligns strongly with other WSPA models (e.g. Transform-Us!, [41]) which highlight the influence of interpersonal and diverse organisational contexts on stakeholder behaviour and practice [42]. The importance of strong relationships to build ownership and shared responsibility, and specifically the role of trust has previously been highlighted as underpinning the collaborative processes through which these groups co-design, implement, co-adapt, and scale up an WSPA and its supporting implementation strategies [43]. The findings underline that without adequate time (Practice recommendation 3), commitment (Practice recommendation 4), support (Practice recommendation 2), and professional learning (Practice recommendation 8), school staff are unlikely to have the confidence or capacity to embed physical activity amidst competing priorities [20]. This supports a shift away from top-down implementation towards relational and network-based approaches, where learning is shared through communities of practice, school-to-school networks, and embedded research partnerships (Research recommendation 5). These activities are key in facilitating transdisciplinary learning with enhanced outcomes by integrating diverse perspectives, including those from non-academic stakeholders such as community members and policymakers [44] (Policy recommendation 3 & 4).

The middle layer of the model represents the four interdependent system conditions that act as mechanisms to produce change over time (i.e. partner engagement and collaboration, evidence base and action orientation, knowledge mobilisation, support and capacity building). While these are long standing themes of the Health Promoting Schools framework [45], the framing of these as sustainability mechanisms emphasises their role as applied system levers for ongoing professional and organisational adaptation [46]. Evidence from the CAS programme illustrates how these conditions function dynamically in practice. Sustained implementation was underpinned by strong relational infrastructure, including cross-school and cross-sector collaboration facilitated through communities of practice and the role of the CAS Champions (individuals with specialist knowledge and experience of the educational system and the promotion of physical activity to support the delivery of CAS) [18]. Thus, highlighting partner engagement not simply as stakeholder involvement, but as a mechanism that builds combined agency across the system (Practice recommendation 7).

Finally, the outer ring represents the macro-level forces (e.g. accountability structures and mandated physical activity), that shape what is possible inside schools. Current accountability structures were frequently described as fragmented and contributing to confusion, limiting schools’ ability to prioritise physical activity in meaningful ways. While mandating physical activity within curricula (Policy recommendation 1) or accountability frameworks (Policy recommendation 6) was viewed as a potential lever for prioritisation and equity, our findings suggest that mandates must be accompanied by aligned guidance, resources (Practice recommendation 6), and professional development (Practice recommendation 8) to avoid superficial compliance. Subsequently, we support previous research which has also called for coherence across education, health, and sport policy (Policy recommendation 3), so that expectations placed on schools are realistic, supported, and mutually reinforcing [47]. Embedding WSPA within accountability frameworks was seen as necessary, but only if evaluation approaches are proportionate, context-sensitive (Research recommendation 2), and supportive of learning rather than punitive (Research recommendation 3, Policy recommendation 10). Our findings indicate the need for accountability systems and research and evaluation that prioritise process, culture change, and equity in addition to outcomes (Research recommendation 4). This requires supporting schools as learning organisations within a wider system of shared responsibility [48].

### Strengths and limitations

This is the first study to co-design priority areas for action to advance WSPA approaches across the interconnected domains of research, policy, and practice. It builds on previous work by drawing on the expertise and lived experience of a large and diverse stakeholder group from multiple cultural and country contexts (local, regional, and national) across Europe and beyond. Moreover, it includes representatives from WSPA programmes in countries such as Estonia and Finland that have led national implementation of evidence-informed WSPA programmes. By integrating multiple perspectives in this way, this study advances our understanding of how to enable system-level change in schools that is generated and can be sustained within educational systems.

Although the use of a convenience sample drawn from delegates attending the #WSPA2024 conference may have introduced some bias, it provided a large amount of information rich participants, this was reflected in the quality of the dialogue during the workshops. Furthermore, the results suggest some common experience which is transferable to country contexts, and complements other work completed in this area (see [49]).

However, despite strong engagement during the face-to-face co-design process, the response rate for reviewing and ratifying the drafted recommendations was relatively low. The volume of data collected meant that the time required for recording and synthesis resulted in the ratification process taking place approximately six months after the initial data collection. While engagement during the conference itself was high, it is likely that some momentum was lost post-event, which may account for the reduced participation at this stage. Future research should consider strategies to maintain stakeholder engagement across longer timeframes and aim to replicate and expand this approach with stakeholders from a broader range of regions, particularly low- and middle-income countries, to test and refine the global applicability of these insights.

## Conclusions

WSPA approaches must be positioned as a long-term systems change agenda, requiring aligned policy, flexible practice, and innovative research approaches. Embedding physical activity across curricula, governance, and culture is not only a health priority but also a strategy for enhancing learning, wellbeing, and equity. The recommendations generated from the WSPA recommendations offer a timely platform to argue for multi-sector collaboration, co-production, and investment in scalable, context-sensitive solutions. We call on all sectors to take steps to action these recommendations and fulfil the potential of WSPA approaches for children’s health and wellbeing.

## Supplementary Information

Supplementary Material 1

## Data Availability

The dataset that generated and analysed during the current study are available from the corresponding author on reasonable request.

## Abbreviations

CAS: Creating Active Schools
MVPA: Moderate to vigorous physical activity
WSPA: Whole–school physical activity
#WSPA2024: The International Whole School Physical Activity Conference 2024
